# Mental health literacy questionnaire-short version for adults (MHLq-SVa): validation study in China, India, Indonesia, Portugal, Thailand, and the United States

**DOI:** 10.1186/s12888-022-04308-0

**Published:** 2022-11-16

**Authors:** Luísa Campos, Pedro Dias, Marisa Costa, Laura Rabin, Rona Miles, Sumi Lestari, Rania Feraihan, Neera Pant, Natthaphansan Sriwichai, Waraporn Boonchieng, Luxi Yu

**Affiliations:** 1 Research Centre for Human Development, Universidade Católica Portuguesa, R. Diogo Botelho, 1327, 4169-005 Porto, Portugal; 2 Faculty of Education and Psychology, Universidade Católica Portuguesa, R. Diogo Botelho, 1327, 4169-005 Porto, Portugal; 3 Department of Psychology, University of the Azores, Rua da Mãe de Deus, 9500-321 Ponta Delgada, Portugal; 4 Department of Psychology, Brooklyn College of The City University of New York, 2900 Bedford Avenue, 11210 Brooklyn, NY USA; 5 Psychology, Faculty of Social and Political Sciences, University of Brawijaya, 65145 St.Veteran, Malang City, East Java, Indonesia; 6 Gargi College, University of Delhi, Siri Fort Road, 110049 New Delhi, India; 7 Thanyarak Chiangmai Hospital, 182 Moo 7, Ki Lek, Mae Rim, Chiang Mai, 50180 Thailand; 8 Faculty of Public Health, Chiang Mai University, Chiang Mai, Thailand; 9 Medical Psychology Teaching and Research Section School of Medical Humanities, Guizhou Medical University, Guizhou, China

**Keywords:** Mental Health literacy, Questionnaire, Undergraduate students, Short version, Validation, Portugal, USA, China, Thailand, India, Indonesia

## Abstract

**Background:**

Mental Health Literacy (MHL) has become a focus of research in recent decades, as a prerequisite for early identification and intervention for mental health problems. Although several instruments have been developed for assessing MHL, there is a need for brief and psychometrically sound measures to capture important aspects of MHL in large and diverse adult samples. The present study aimed to: (1) provide a revised and shorter version of a previously validated questionnaire for assessing MHL; and (2) examine the psychometric properties of the MHLq-SVa in student samples from six different countries (China, India, Indonesia, Portugal, Thailand, and United States).

**Methods:**

The study involved 2180 senior school and undergraduate students, aged between 17 and 25 years old, from China, India, Indonesia, Portugal, Thailand, and the United States. Participants responded to the Mental Health Literacy Questionnaire for young adults (MHLq-ya), in their native language, following its translation and adaptation for each culture. The MHLq-ya comprises 29 items, organized into four dimensions: Knowledge of mental health problems; Erroneous beliefs/stereotypes; First-aid skills and help-seeking behavior; Self-help strategies. Confirmatory factor analyses and internal consistency analyses were performed on the combined data.

**Results:**

Data from the different countries supported a shorter version of the questionnaire (MHLq-SVa), composed of 16 items that fit with previously defined dimensions. Internal consistency and between-factor correlations further supported the adequacy of the instrument’s psychometric properties.

**Conclusion:**

The study provided preliminary support for the construct validity and reliability of the MHLq-SVa as a measure for assessing MHL in young adults from six different countries and languages. Future studies are needed to further validate the measure and undertake multicultural comparisons of MHL in diverse samples from around the globe.

## Background

According to the Global Burden of Disease Study, in 2019 approximately 792 million people presented mental health problems, a number that rises to 970 million people if substance use is included [1]. In recent years, several studies [2, 3] have reported an increase in the prevalence of these problems. The estimated prevalence rates of mental health problems and substance use vary widely by region and country: for example, in the US, the estimated rate was 16.9%; in Portugal, 18.5%; in eastern countries, rates tend to be lower (e.g., Thailand, 12.0%; China, 11.3%; India, 13.7%; Indonesia, 10.7%) [4]. The COVID-19 pandemic led to a global increase of mental health problems, particularly stress, depressive and anxious symptoms [5, 6], suggesting an increased need for mental health assessment as well as the provision of services and efficacious interventional programmes.

The increasing prevalence of mental health problems globally suggests the need to adopt preventative measures, as early detection of signs and symptoms increases the probability of success in terms of treatment outcomes [7–13]. One relevant resource for the prevention of mental health problems is Mental Health Literacy (MHL), a multifactorial construct encompassing: (1) knowledge related to the prevention of mental health problems; (2) recognition of signs and symptoms (i.e., being able to identify the onset of problem development); (3) identification of available options and treatments; and (4) knowledge of tailored self-help strategies and first-help skills to support others who are developing and/or present with mental health problems [9]. Low levels of MHL have been found to be related to difficulties in recognizing mental health problems (whether in oneself or in others) [12], delay in seeking help, inadequate use of resources and action strategies, as well as communication difficulties with health professionals and lower adherence to treatments [2, 14–17].

Research has examined the role of variables that potentially influence MHL levels, namely gender [7, 18], proximity to someone with mental health problems [3, 19–22] and culture [23, 24]. The results of these studies indicate that young female adults [3, 25–28] and people with proximity to people with mental health problems [7, 15, 18, 29–32] tend to present higher levels of MHL. With regard to culture and its influence on MHL, factors such as personal beliefs, religion, language, cultural diversity and subjective experience seem to influence knowledge about mental health problems [9, 23, 33, 34]. Studies that compared the MHL levels of participants from different countries and regions found that participants from western and developed regions presented higher levels of MHL, in comparison to participants from developing regions [8, 35].

Several instruments have been developed to assess MHL (see reviews [27, 36]), some of them focusing on specific dimensions (e.g., knowledge; stigmatizing perceptions) or specific mental health problems or diagnoses (e.g., schizophrenia; depression) [7]. Taking into account the updated construct of MHL [9], and limitations of previous measures (e.g., use of time-consuming vignettes, measures limited to specific mental health problems), a new instrument to provide a more up-to-date assessment of this construct was developed for assessing MHL in young people (Mental Health Literacy questionnaire – young people form; MHLq-YP; [7]). In 2018, Dias and collaborators adapted this questionnaire for young adults - the Mental Health Literacy questionnaire-young adult form; MHLq-YA. The MHLq-YA includes 29 items, rated on a five-point Likert-type scale, organized into four dimensions: (1) knowledge of mental health problems, (2) erroneous beliefs/stereotypes, (3) first aid skills and help seeking behaviour, and (4) self-help strategies. The preliminary study of this instrument’s psychometric properties showed appropriate levels of validity and internal consistency [18].

Given the increasing need to assess MHL among young adults and given time constraints when undertaking such assessments, the current study aimed to expand the analysis of the psychometric properties of the MHLq-YA to provide a revised and shorter version. In addition, we examined the psychometric properties of this short version in student samples from six different countries (China, India, Indonesia, Portugal, Thailand, and United States).

## Methods

### Participants

The study involved 2180 senior school and undergraduate students, aged between 17 and 25 years old, from six different countries – China, India, Indonesia, Portugal, Thailand, and the United States.

The sample from China included 496 undergraduates (63% female), aged from 17 to 22 years (M = 19.00; SD = 0.84). The sample from India included 284 undergraduate students (63% female), aged between 18 and 25 years (M = 20.85; SD = 1.85). The Indonesian sample included 197 undergraduate students (63% female), aged between 18 and 23 years (M = 19.08; SD = 1.28). The Portuguese sample included 382 undergraduate students (53% female), aged between 17 and 25 years (M = 21.21, SD = 2.15). The Thai sample included 385 undergraduate students (70% female), aged between 18 and 24 years (M = 20.26; SD = 1.30). The United States sample included 436 undergraduate students aged between 18 and 25 years (M = 20.60; SD = 1.99; 60% female).

### Procedure

Prior to data collection, the MHLq-YA was translated into the predominant language of each country, following the guidelines for translating and adapting items [37]: (1) translation from English or Portuguese by a bilingual translator; (2) back-translation by a different bilingual translator; (3) pilot testing of items, through think aloud procedures (Thailand) or comprehension rating using a 3-point scale questionnaire (China); (4) semantic comparison of the translation and back-translation; (5) review by experts in psychometrics and linguistics (India, Indonesia, and Thailand); (6) analysis of the translated version by the Portuguese research team.

Participants’ recruitment occurred through in person contact with students (Portugal, US, and Thailand) and through online dissemination (China, India, Indonesia, Portugal, Thailand, and US).

The study followed ethical guidelines in each country with all participants providing written informed consent. The sociodemographic and MHLq-YA forms were self-administered using online platforms (India, Indonesia,Thailand), in-person paper-and pencil questionnaires (China, US), or both (Portugal). Data collection occurred both prior to (Portugal, Thailand, and US) and during (China, Thailand, India and Indonesia) the COVID-19 pandemic.

### Instruments

The protocol included a sociodemographic section, comprising self-reported questions about gender and age, and the MHLq-YA.

#### Mental Health literacy questionnaire – young adult form (MHLq-YA)

The original version of MHLq-YA [18] included 29 items, developed to measure MHL on four dimensions: (1) knowledge of mental health problems (e.g., “A person with depression feels very miserable.”; “People with schizophrenia usually have delusions.”), (2) erroneous beliefs/ stereotypes (e.g., “Mental disorders don’t affect people’s behaviors.”; “People with mental disorders belong to low-income countries.”, (3) help-seeking and first aid skills (e.g., “If I had a mental disorder, I would seek my relatives’ help.”; “If someone close to me had a mental disorder, I would encourage her/him to look for a psychologist.”, and (4) self-help strategies (e.g., “Physical exercise contributes to good mental health.”; “Sleeping well contributes to good mental health.”). Participants were asked to rate each item, ticking the option that indicates how much they agree or disagree, using a five-point scale (ranging from 1 = Strongly Disagree to 5 = Strongly Agree) was used to respond to the items. Cronbach’s Alpha for the total scale in the adaptation study was 0.84.

### Data analysis

Considering the preliminary evidence from the MHLq-YA study [18], first we performed a confirmatory factor analysis (CFA) on the original structure with the Portuguese sample. For CFA analyses, we used AMOS software (SPSS Inc, Chicago, IL, version 27.0) and the estimation method chosen was maximum likelihood (ML). Following the theoretical recommendations, to evaluate the goodness of fit of models, we used the following global indeces: Chi-square (X2) and Chi-square difference (X2/gl); Non-Normed Fit Index (NNFI) and Comparative Fit Index (CFI); and Root Mean Squared Error of Approximation (RMSEA). The standard value for CFI is equal to or greater than 0.95, and lower or equal to 0.08 for RSMEA [38–40]. For the analysis of local adjustment and item elimination, in addition to factor loadings, we also considered the magnitude of the Squared Multiple Correlation Coefficient (R2), the variances and covariance, and the amount of error associated [38, 39]. Taking into account the limitations in several items identified in the exploratory study [18], and the above mentioned local adjustment indices, elimination of items would be considered, resulting in a shortened version of MHLq-YA to be tested with the samples from China, India, Indonesia, Thailand, and the United States.

For the calculation of total score, items from “erroneous beliefs/stereotypes” dimension were reverse-scored. Internal consistency was analysed in all samples through Cronbach’s Alpha and McDonald’s Omega [41], as well as the interrelation among subscales, using SPSS (SPSS Inc, Chicago, IL, version 27.0). McDonald’s Omega coefficient provides more accuracy to the approximation of internal reliability, when compared to Cronbach’s Alpha. Standard values stipulate that McDonald’s Omega values above 0.70 are acceptable, and, for Cronbach’s Alpha, a coefficient of 0.60 is the cut-off for a measure to be considered internally reliable [42]. Statistical significance was set at p < .05.

## Results

### Confirmatory factor analyses

The construct validity of the MHLq-YA was tested by performing a CFA with the Portuguese sample. Considering the previous results of the exploratory study of psychometric properties [18], local adjustment indicators, as well as the global adjustment, 13 items were removed from the model, namely: MHLq2, MHLq3, MHLq4, MHLq5, MHLq9, MHLq10, MHLq12, MHLq11, MHLq14 MHLq18, MHLq21, MHLq23 and MHLq24. Also, the modification indices specified the correlations between the errors of MHLq06 and MHLq13 items, the MHLq08 and MHLq17 items, as well as MHLq01 and MHLq19.

The revised version of MHLq-YA, now named Mental Health Literacy questionnaire- Short Version for adults (MHLq-SVa), fit well to the Portuguese data (X2(95) = 153.17 × 2/gl = 1.62 ; CFI = 0.95; NNFI = 0.95 RMSEA = 0.040). At the level of local adjustment, the factorial weights were globally high, ranging between 0.50 and 0.97 (Table 1).

The quadri-dimensional model showed similar goodness-of-fit indices in US (X2 (95) = 206.547 × 2/gl = 2.17; CFI = 0.97; NNFI = 0.96; RMSEA = 0.052), China (X2 (95) = 186.190 × 2/gl = 1.96; CFI = 0.95; NNFI = 0.94; RMSEA = 0.044), Thailand (X2 (95) = 180.768 × 2/gl = 1.90; CFI = 0.95; NNFI = 0.94; RMSEA = 0.048), India (X2 (95) = 257.482 × 2/gl = 2.71; CFI = 0.88; NNFI = 0.85; RMSEA = 0.072) and Indonesia (X2 (95) = 183.044 × 2/gl = 1.92; CFI = 0.95; NNFI = 0.94; RMSEA = 0.051). The model also showed a good local adjustment in these five countries (Table 1), with items loadings ranging from 0.20 to 0.93 and globally good R^2^ values (above 0.30). The standardized loadings revealed some concerns in two countries, regarding items MHLq13 (Indonesia), MHLq15, and MHLq27 (India).

**Table 1:** Standardised Factor Loadings, Standardised Error and Squared Multiple Correlations for China, India, Indonesia, Portugal, Thailand, and US.

	Portugal			US			China			Thailand			India					Indonesia				
**Dimension/ Items**	**SL**	**SE**	**R** ^**2**^	**SL**	**SE**	**R** ^**2**^	**SL**	**SE**	**R** ^**2**^	**SL**	**SE**	**R** ^**2**^	**SL**		**SE**	**R** ^**2**^		**SL**		**SE**		**R** ^**2**^
Knowledge of mental health problems																						
MHLq25	0.57	0.12	0.33	0.74	0.13	0.54	0.71	0.12	0.53	0.63	0.09	0.42	0.60		0.20	0.36		0.58		0.11		0.34
“Mental disorders affect people’s thoughts”																						
MHLq27	0.57	0.15	0.33	0.72	0.12	0.53	0.40	0.12	0.15	0.52	0.12	0.27	0.25		0.18	0.07		0.48		0.17		0.23
“A person with schizophrenia may see and hear things that nobody else sees and hears”																						
MHLq20	0.54	0.14	0.29	0.69	0.12	0.48	0.46	0.12	0.19	0.63	0.11	0.39	0.37		0.18	0.14		0.60		0.14		0.37
“One of the symptoms of depression is the loss of interest or pleasure in most things”																						
MHLq28	0.53	0.13	0.28	0.66	0.11	0.44	0.65	0.11	0.40	0.65	0.10	0.44	0.68		0.19	0.46		0.40		0.15		0.16
“Highly stressful situations may cause mental disorders”																						
MHLq16	0.55		0.30	0.59		0.35	0.54		0.28	0.62		0.38	0.41			0.17		0.56				0.31
“Changes in brain function may lead to the onset of mental disorders”																						
MHLq22	0.51	0.15	0.26	0.48	0.10	0.23	0.48	0.12	0.24	0.59	0.09	0.34	0.50		0.20	0.25		0.60		0.14		0.36
“The symptoms’ length is one of the important criteria for the diagnosis of a mental disorder”																						
Erroneous beliefs/stereotypes																						
MHLq15	0.68		0.46	0.86		0.74	0.39		0.05	0.64		0.25	1.96			3.83		0.82				0.67
“Only adults have mental disorders”																						
MHLq13	0.54	0.17	0.29	0.69	0.06	0.48	0.70	0.73	0.07	0.81	0.24	0.26	0.20		0.17	0.04		1.00		0.15		0.99
“Mental disorders don’t affect people’s feelings”																						
MHLq06	0.59	0.15	0.35	0.72	0.06	0.52	0.48	0.57	0.05	0.73	0.23	0.29	0.25		0.21	0.06		0.81		0.15		0.66
“Mental disorders don’t affect people’s behaviors”																						
Help-seeking and first aid skills																						
MHLq8	0.68		0.46	0.82		0.67	0.92		0.66	0.76		0.50	0.79			0.62		1.04				1.07
“If I had a mental disorder, I would seek for a psychologist’s help”																						
MHLq17	0.97	0.18	0.94	0.94	0.06	0.88	0.72	0.05	0.36	0.76	0.08	0.52	0.88		0.10	0.77		0.77		0.05		0.59
“If someone close to me had a mental disorder, I would encourage her/him to see a psychiatrist”																						
MHLq29	0.62	0.25	0.38	0.88	0.07	0.80	0.79	0.07	0.82	0.82	1.00	0.73	0.68		0.10	0.46		0.75		0.13		0.56
“If I had a mental disorder, I would seek for a psychiatrist’s help”																						
Self-help strategies																						
MHLq7	0.62	0.17	0.39	0.79	0.07	0.62	0.64	0.07	0.41	0.70	0.16	0.48	0.64		0.11	0.41		0.72		0.64		0.51
“Sleeping well contributes to a good mental health”																						
MHLq19	0.58	0.22	0.34	0.79	0.07	0.61	0.68	0.09	0.46	0.76	0.19	0.59	0.66		0.10	0.43		0.63		0.64		0.40
“A balanced diet contributes to a good mental health”																						
MHLq1	0.49	0.16	0.24	0.78	0.08	0.59	0.34	0.10	0.12	0.58	0.16	0.37	0.54		0.10	0.29		0.49		0.44		0.24
“Physical exercise contributes to a good mental health”																						
MHLq26	0.50		0.25	0.73		0.54	0.72		0.51	0.50		0.25	0.60			0.36		0.32				0.10
“Doing something enjoyable contributes to a good mental health”																						

SL – Standardised Loadings, SE – Standardised Error, R^2^ – Squared Multiple Correlations

### Reliability

The reliability of MHLq-SVa for each country is reported in Table 2. Both Alpha and Omega coefficients for each dimension and total score were close, ranging from 0.59 to 0.93.

**Table 2:** Descriptive Statistics of MHLq-SVa Dimensions, Cronbach’s alpha and McDonald’s Omega

		Portugal		US		China		Thailand		India		Indonesia	
MHLq Dimensions	Items	α	Ω	α	Ω	α	Ω	α	Ω	α	Ω	α	Ω
Knowledge of mental health problems	6	0.72	0.71	0.83	0.83	0.72	0.71	0.79	0.78	0.66	0.64	0.76	0.75
Erroneous beliefs/stereotypes	3	0.67	0.67	0.83	0.83	0.59	0.65	0.76	0.76	0.72	0.72	0.89	0.89
Help-seeking and first aid skills	3	0.69	0.75	0.91	0.92	0.81	0.83	0.81	0.82	0.81	0.82	0.84	0.87
Self-help strategies	4	0.66	0.68	0.86	0.86	0.67	0.66	0.74	0.75	0.72	0.72	0.60	0.67
MHLq Total	16	0.82	0.80	0.93	0.93	0.80	0.77	0.86	0.84	0.81	0.73	0.82	0.78

α - Cronbach’s Alpha ; Ω - McDonald’s Omega

The correlations between dimensions and the total score of MHLq-SVa (Table 3) ranged from − 0.29 to 0.92. As expected, considering the nature of the items included in dimension 2 (erroneous beliefs/stereotypes), negative correlations were found between this dimension and the other three dimensions and the total score in all countries, except India.

**Table 3:** Means, Standard Deviations and Correlations Among MHLq Dimensions and Total Score

			Portugal				
	M(SD)	2.	3.		4.		5.
1. Knowledge of mental health problems	4.05 (.48)	− .46**	.22**		.51**		.83**
2. Erroneous beliefs/stereotypes	1.69 (.68)		− .17**		− .39**		− .71**
3. Help-seeking and first aid skills	4.10 (.66)				.21**		.53**
4. Self-help strategies	4.29 (.49)						.73**
5. MHLq Total	4.17 (.39)						
			**US**				
	M(SD)	2.	3.	4.		5.	
1. Knowledge of mental health problems	3.86 (.85)	− .68**	.54**	.76**		.92**	
2. Erroneous beliefs/stereotypes	1.71 (1.07)		− .39**	− .65**		− .80**	
3. Help-seeking and first aid skills	3.77 (1.18)			.54**		.73**	
4. Self-help strategies	3.95 (.99)					.89**	
5. MHLq Total	3.94 (.83)						
			**China**				
	M(SD)	2.	3.	4.		5.	
1. Knowledge of mental health problems	3.92 (.55)	− .29**	.25**	.41**		.82**	
2. Erroneous beliefs/stereotypes	1.74 (.64)		− .09	− .22**		− .55**	
3. Help-seeking and first aid skills	4.29 (.67)			.43**		.60**	
4. Self-help strategies	4.58 (.45)					.71**	
5. MHLq Total	4.21 (.39)						
			**Thailand**				
	M(SD)	2.	3.	4.		5.	
1. Knowledge of mental health problems	3.98 (.51)	− .33**	.52**	.46**		.85**	
2. Erroneous beliefs/stereotypes	1.82 (.72)		− .28**	− .20**		− .61**	
3. Help-seeking and first aid skills	4.21 (.63)			.37**		.72**	
4. Self-help strategies	4.23 (.53)					.69**	
5. MHLq Total	4.13 (.42)						
			**India**				
	M(SD)	2.	3.	4.		5.	
1. Knowledge of mental health problems	3.89 (.62)	.00	.42**	.58**		.86**	
2. Erroneous beliefs/stereotypes	2.24 (1.12)		− .14*	− .08		− .08	
3. Help-seeking and first aid skills	4.41 (.84)			.58**		.76**	
4. Self-help strategies	4.44 (.66)					.85**	
5. MHLq Total	4.18 (.56)						
			**Indonesia**				
	M(SD)	2.	3.	4.		5.	
1. Knowledge of mental health problems	4.12 (.55)	− .29**	.32**	.37**		.75**	
2. Erroneous beliefs/stereotypes	2.36 (1.41)		− .16*	− .21**		− .75**	
3. Help-seeking and first aid skills	4.30 (.71)			.20**		.54**	
4. Self-help strategies	4.34 (.52)					.58**	
5. MHLq Total	4.12 (.50)						

*p < .05; ** p < .01.

## Discussion

The current study aimed to provide evidence of the psychometric properties of a shorter version of the MHLq-YA in six different countries (Portugal, US, China, Thailand, India, and Indonesia).

Following the preliminary study of the psychometric properties of the MHLq-YA [18], where some items presented psychometric issues (e.g., items loading simultaneously in two factors; acceptable, but low loading values), a CFA was conducted with the Portuguese sample, suggesting the exclusion of 13 items. This resulted in a shorter version of the measure, with 16 items organized in four dimensions – Knowledge of mental health problems; Erroneous beliefs/stereotypes; Help-seeking and first aid skills; Self-help strategies – in line with the most recent definition of the MHL construct [9]. This shortened version of MHLq-YA was tested with data from five countries (US, China, Thailand, India, Indonesia). The data from these different countries globally fit the four-factor model tested. The interrelations between factors and total score confirmed the questionnaire’s consistency, with significant contributions of each dimension to the latent construct – MHL. According to the reference values [42], the internal consistency was globally acceptable in each country. The US data shown the highest internal consistency values. This revised version of the MHLq showed a good psychometric structure to be used as an assessment tool of MHL in six countries.

This study has two major strengths. First, it allowed for the development of a shorter version of a questionnaire for assessing MHL in young adults, which will be easier to administer, less time consuming to score, and less burdensome for participants. Second, this was the first time that this questionnaire was tested in different cultures, from distinct regions (Europe, North America, and Asia), in languages spoken in countries with large populations, facilitating future research focused on multicultural comparisons of MHL. There are also limitations. The sample sizes of each country varied significantly, potentially compromising comparative analyses, such as measurement invariance. Data collection procedures (paper-pencil vs. online) and timing (pre and during COVID-19 pandemic) differed between countries. The fact that some data were collected during the pandemic could have influenced participants’ responses, since mental health awareness is thought to have increased during this period (e.g., [43,44]). The difference in data collection procedures could have also affected data comparison between countries, since online and paper-pencil administration could result in different responses, particularly regarding the knowledge dimension, as answers to these items could be found online.

Future studies should address and overcome the limitations stated above, but also contribute to the strengthening and applicability of this instrument. First, considering the concerns regarding the factor loadings of some items, as well as the non-significant correlation between Factor 2 and Factors 1 and 3 in India, new data collection and analyses should be developed in India and Indonesia, in order to explore the need to revise item translation or make cultural adjustments. Second, other instruments should be considered, not only to control social desirability, but also to further study the MHL construct by means of examining concurrent validity (e.g., Mental Health Literacy Scale; [45]). Third, data collection procedures (online vs. paper-pencil) should be compared in order to examine possible differences between them. Fourth, data collection should be extended to more heterogeneous groups of participants (e.g., different age groups, different educational backgrounds, clinical samples), assuring equivalent sample sizes. Fifth, it would be interesting to test the psychometric properties of this measure with samples from other countries and languages, from other regions, such as South America and Africa. Sixth, using larger samples, measurement invariance should also be examined. Finally, a multi-method approach could be used in future research, highlighting the extent to which perceptions are consistent with others’ reports in different cultures.

## Conclusion

Preliminary validation of the MHLq-SVa suggests that it is a valid and reliable measure for assessing MHL in young adults from six different countries and languages (Portugal – Portuguese, US – English, China – Chinese, Thailand – Thai, India - Hindi, and Indonesia – Indonesian). Future studies are needed to test measurement invariance and other relevant psychometric properties, allowing multicultural comparisons of MHL.

## Data Availability

The datasets used and/or analysed during the current study are available from the corresponding author on reasonable request.

## Abbreviations

CFI: Comparative Fit Index
CFA: Confirmatory Factor Analysis
ML: Maximum Likelihood
MHL: Mental Health Literacy
MHLq-YA: Mental Health Literacy questionnaire for young adults
MHLq-SVa: Mental Health Literacy questionnaire – Short Version for adults
NNFI: Non-Normed Fit Index
RMSEA: Root Mean Squared Error of Approximation
R2: Squared Multiple Correlation Coefficient.
